# Smart Diagnosis of Urinary Tract Infections: is Artificial Intelligence the Fast-Lane Solution?

**DOI:** 10.1007/s11934-023-01192-3

**Published:** 2023-12-19

**Authors:** Nithesh Naik, Ali Talyshinskii, Dasharathraj K. Shetty, B. M. Zeeshan Hameed, Rano Zhankina, Bhaskar K. Somani

**Affiliations:** 1 Department of Mechanical and Industrial Engineering, Manipal Institute of Technology, Manipal Academy of Higher Education, Manipal, 576104 Karnataka India; 2 Department of Urology, Astana Medical University, Astana, 010000 Kazakhstan; 3 Department of Data Science and Engineering, Manipal Institute of Technology, Manipal Academy of Higher Education, Manipal, 576104 Karnataka India; 4 Department of Urology, Father Muller Medical College, Mangalore, 575002 Karnataka India; 5 iTRUE-International Training and Research in Urology and Endourology, Manipal, 576104 Karnataka India; 6 Department of Urology, University Hospital Southampton NHS Trust, Southampton, SO16 6YD UK

**Keywords:** Artificial intelligence, Smart diagnosis, Urology, Tract infection, Diagnosis

## Abstract

**Purpose of Review:**

Artificial intelligence (AI) can significantly improve physicians’ workflow when examining patients with UTI. However, most contemporary reviews are focused on examining the usage of AI with a restricted quantity of data, analyzing only a subset of AI algorithms, or performing narrative work without analyzing all dedicated studies. Given the preceding, the goal of this work was to conduct a mini-review to determine the current state of AI-based systems as a support in UTI diagnosis.

**Recent Findings:**

There are sufficient publications to comprehend the potential applications of artificial intelligence in the diagnosis of UTIs. Existing research in this field, in general, publishes performance metrics that are exemplary. However, upon closer inspection, many of the available publications are burdened with flaws associated with the improper use of artificial intelligence, such as the use of a small number of samples, their lack of heterogeneity, and the absence of external validation. AI-based models cannot be classified as full-fledged physician assistants in diagnosing UTIs due to the fact that these limitations and flaws represent only a portion of all potential obstacles. Instead, such studies should be evaluated as exploratory, with a focus on the importance of future work that complies with all rules governing the use of AI.

**Summary:**

AI algorithms have demonstrated their potential for UTI diagnosis. However, further studies utilizing large, heterogeneous, prospectively collected datasets, as well as external validations, are required to define the actual clinical workflow value of artificial intelligence.

## Introduction

**Fig. 1:**
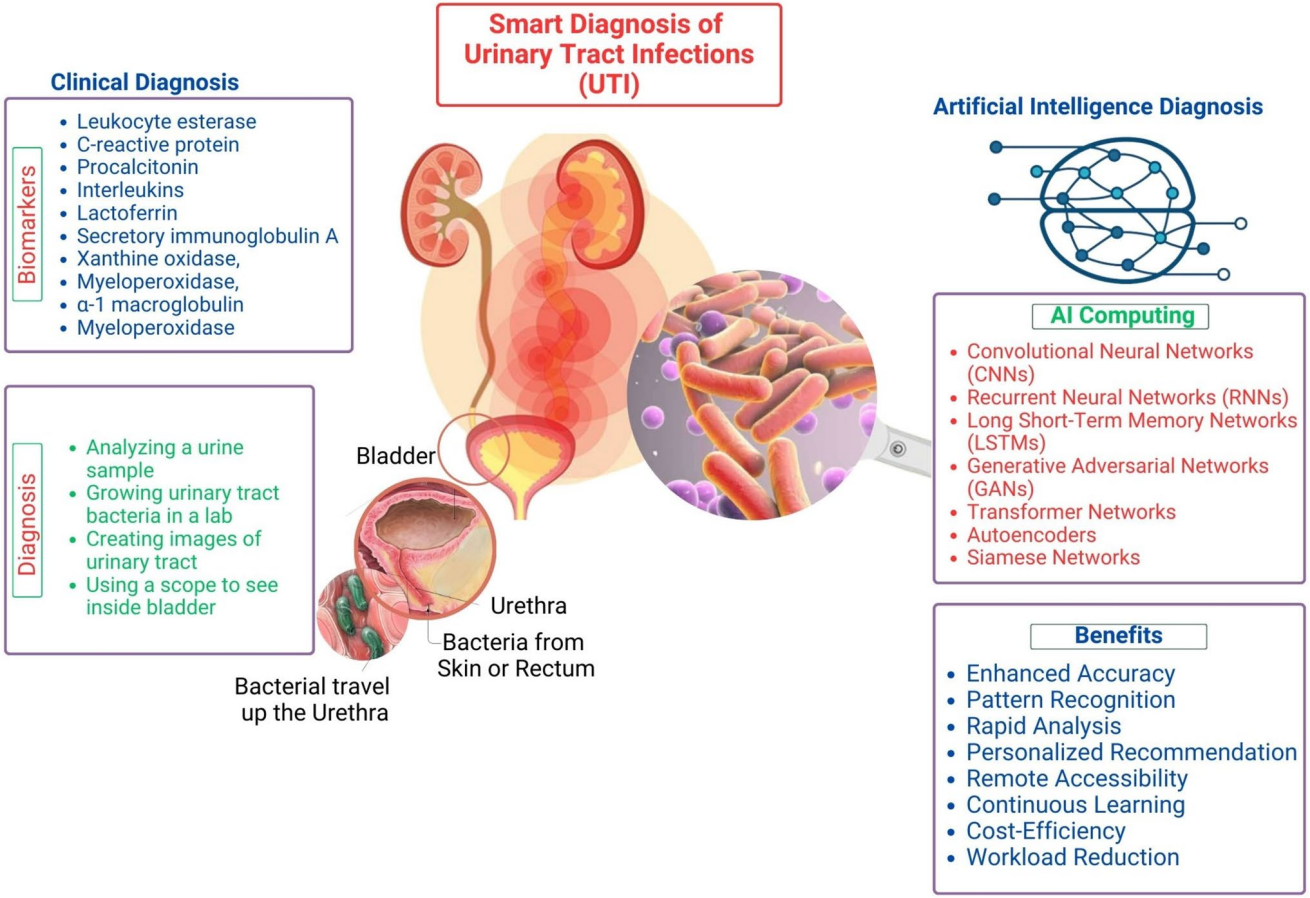
Artificial intelligence (AI)–based treatment and diagnosis of UTIs

Urinary tract infections (UTIs) are the most prevalent outpatient illnesses and affect about 50% of the population at some point in their lives [1]. The incidence of UTIs is increasing with age, and close to 10% of postmenopausal women indicate that they had a UTI in the previous year [2]. Moreover, it is a frequent emergency department (ED) diagnosis with reportedly high diagnostic inaccuracy [3]. According to clinical criteria alone, the diagnosis of UTI has a diagnostic error rate of approximately 33% [4]. Different classification systems for UTI exist. Despite this diversity, defining UTI is reduced to the presence of bacteria in the urinary tract accompanied by related symptoms and dividing UTI into noncomplicated and complicated groups, with the latter leading to severe consequences, such as urosepsis, if untreated [5••]. The algorithm for diagnosing patients with suspected UTIs consists of several stages; each of the subsequent ones allows for more reasoned further diagnostics to make the correct diagnosis. Figure 1 shows the artificial intelligence (AI)–based treatment and diagnosis of UTIs.

Liquid-based laboratories, such as urine analyses with microscopy and culturing, represent the standard for the initial diagnosis of UTI to suspect pathologies of the urinary system and to specify indications for an instrumental approach [6•, 7]. Currently, it is well known that UTI-associated urine changes could be present in non-infection urinary tract pathologies, leading to decreased urine microscopy accuracy [8]. Moreover, urine culture suffers from several shortcomings, such as being time-consuming and highly susceptible to contamination, leading to incorrect antibiotic prescription, overutilization, antibiotic resistance, and postponed treatment [9]. The use of AI in the medical industry has grown and expanded over time. Among them, the development of intelligent decision-making for clinical medicine is the fastest [10]. Currently, it has already been stated that AI-based models can significantly improve physicians’ workflow when examining patients with UTI [11••]. However, most contemporary reviews focus on examining AI usage with a restricted quantity of data, analyzing only a subset of AI algorithms, or performing narrative work without analyzing all dedicated studies. Given the preceding, the goal of this work was to conduct a mini-review to determine the current state of AI-based systems as a support in UTI diagnosis.

## Material and Methods

### Search Strategy

In July 2023, the systematic publication search was done in several databases, including ACM Digital Library, CINAHL, IEEE Xplore, PubMed, and Google Scholar via Boolean operators with the use of the following terms: “AI,””artificial intelligence,” “UTI,” “urinary tract infection,” “cystitis,” “pyelonephritis,” “prostatitis,” “orchitis,” “epididymitis,” “urine,” “urinalysis,” and “urine culture.”

*Inclusion criteria*: description of the development and validation of AI-based approaches for UTI diagnosis based on clinical and/or laboratory and/or instrumental data, description of the AI model used, presence of performance metrics; publication date within 5 years from the search time; English-written papers; accessibility of full papers.

*Exclusion criteria*: papers not in the English language; papers published more than 5 years ago. Also, papers describing solely the technological aspects of the proposed method without its clinical implementation were excluded.

### Studies Process

Two reviewers (A. T. and N. N.) independently identified all papers. All studies fitting the inclusion criteria were selected for full review. If there was disagreement or discrepancy, the senior author (B. K. S.) made the final decision.

### Data Extraction and Analysis

We reviewed studies and extracted information related to the objective, dataset volume, data used for the training, AI approach with precise classification or networks used, performance metrics, outcomes, and validation type. In papers comparing several AI-based models, the most accurate was included in the table. After investigation of the included papers, we divided the described AI models into basic clinical scenarios where they are supposed to be used. This study was reported according to the Preferred Reporting Items for Systematic Reviews and Meta-Analysis (PRISMA) guidelines.

## Results

Out of the 782 papers that were considered, only 14 studies on AI models in the area of UTIs met the criteria for inclusion (Fig. 1). These can be grouped according to the scenarios the AI models were developed for, namely, (1) diagnosis of uncomplicated UTI and symptoms checkers, (2) diagnosis of complicated UTI, and (3) diagnosis of UTIs in specific population groups. Among models, 12 and two papers described machine and deep learning approaches, respectively. The most popular machine learning model was the artificial neural network (ANN) described in six studies, followed by extreme gradient boosting (XGBoost) (*n* = 3), support vector machine (SVM) (*n* = 1), CatBoost (*n* = 1), and ensemble learning model (ELM) (*n* = 1). Among predictive inputs, demographic parameters were used in 10/14 (71.4%) studies and mostly in the view of age (*n* = 9), gender (9), race, and weight (*n* = 1). Notably, the latter are included in papers with pediatric patients. Anamnesis was considered in 7/14 (50%) papers, namely, history of previous UTIs (*n* = 4), history of previous antibiotic treatment failure (*n* = 1), history of previous urine culture results (*n* = 1), and invasive urethral procedures (*n* = 1). Comorbidities were used in 3/14 (21.4%) studies: diabetes (*n* = 2), pneumonia (*n* = 2), classification of stroke (*n* = 1), and the presence of mixed cerebrovascular disease (*n* = 1). Logically, the last two were used in developing AI decision support for stroke patients. UTI-associated symptoms were included in 7/14 (50%) papers: dysuria (*n* = 4), fever (*n* = 3), suprapubic pain (*n* = 3), frequency and urgency (*n* = 1), pollakiuria (*n* = 1), and urine incontinence (*n* = 1). Urinalysis was used, and prognostic input was provided in 7/14 (50%) papers; two of them included dipstick tests only. When urine microscopy was used, red blood cells (RBC), white blood cells (WBC), bacteria’s presence, nitrites, epithelial cells, and glucose were analyzed in 2, 3, 2, 1, 1, and 1 studies, respectively. The study used urine cloudiness as one of its features. Imaging data were used in 3/14 (21.4%): one study analyzed the cystoscopic appearance of the lower urinary tract, and two papers described ultrasound imaging usage (for estimation of hydronephrosis and vesicoureteral reflux grades, respectively). Also, there were other inputs not related to the abovementioned groups: length of stay (LOS) (*n* = 3), length of urethral catheterization (*n* = 2), immunological urine markers (*n* = 1), ward (*n* = 1), serum creatinine and albumin (*n* = 1 and *n* = 1), glucocorticosteroid use (*n* = 1), and duration of immobility (*n* = 1). Performance metrics, validation type as well, and the abovementioned data arranged to include studies are discussed and presented in the review.

### Uncomplicated UTI AI-Based Diagnosis and Symptom Checkers

**Fig. 2:**
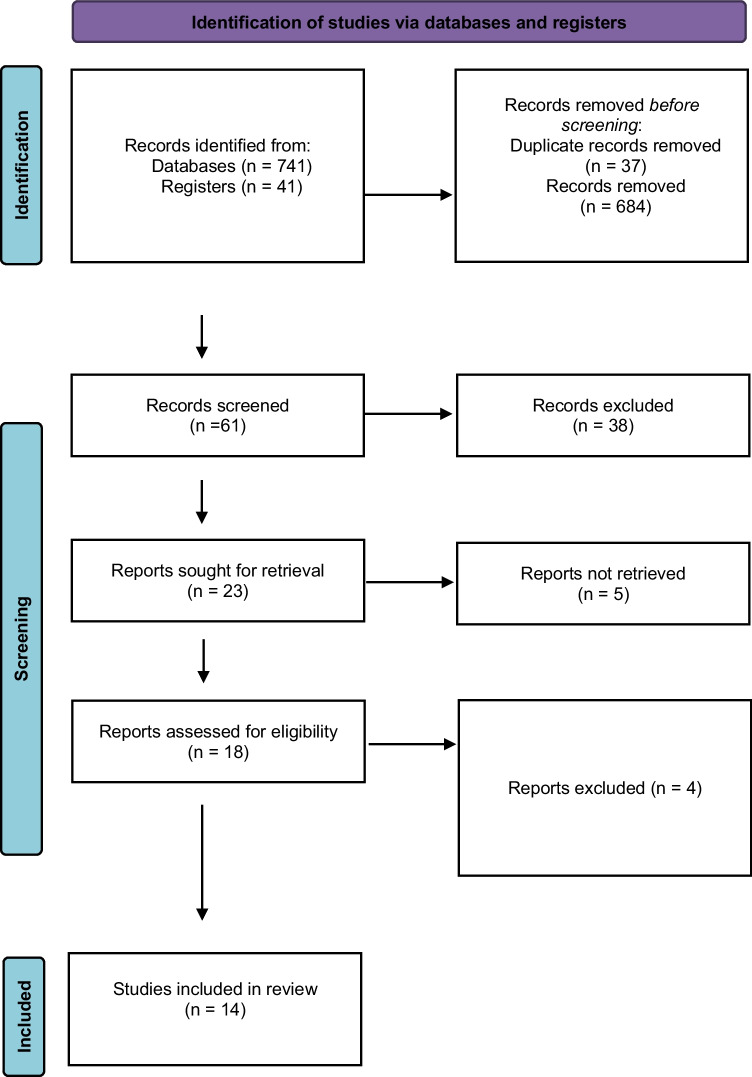
PRISMA flowchart: AI-based approaches for UTI diagnosis

Research on AI-based models for uncomplicated UTI diagnosis and symptom checking is listed in Table 1. The study by Ozkan et al. [12••] sought to determine the accuracy of several artificial intelligence models in predicting the likelihood of cystitis and non-specific urethritis disorders, given similar symptoms from the urinary system. Anamnesis, urinalysis, and ultrasound results from 59 individuals were gathered as a training and validation dataset for the study. Four distinct artificial intelligence techniques were applied: decision trees (DT), random forests (RF), support vector machines (SVM), and artificial neural networks (ANN). When these models were compared, it became evident that ANN had the greatest accuracy for UTI detection, with a result of 98.3%. This ANN model only requires the variables pollakiuria, erythrocyturia, and suprapubic pain to acquire a diagnosis with comparable accuracy to a clinical-based diagnosis (Fig. 2).

**Table 1:** Uncomplicated UTI AI-based diagnosis and symptom checkers

Author	Objective	Dataset (*n*)	AI model	Demographics	Anamnesis	Comorbidity	Symptoms	Urine analysis	Imaging	Other	Performance	Validation
Ozkan et al. [12••]	Prediction of cystitis and non-specific urethritis	59	ANN	-	-	-	Pollakiuria, suprapubic pain	RBC	-	-	Accuracy, 98.3% Sensitivity, 97.7% Specificity, 100%	Internal
Gadalla et al. [13]	Prediction of UTI	183	SVM + RF	-	-	-	-	Cloudiness	-	Immunological markers	AUC, 0.86	Internal
Dhanda et al. [14]	Prediction of UTI in ED	80,859	XGBoost	Age, gender	History of UTI	-	Dysuria, suprapubic pain	Dipstick test results	-	-	AUC, 0.85	External
Arches et al. [15]	Prediction of UTI	65	CNN	-	-	-	-	Dipstick test results	-	-	Accuracy, 96%	Internal

It was demonstrated that the ANN-based model structure could categorize UTIs without the requirement for expensive laboratory testing, ultrasounds, or invasive methods. Hence, it results in a cheaper diagnostic cost and a quicker decision-making process.

The motivation behind Gadalla et al.’s [13] paper is that women with uncomplicated UTI symptoms are frequently treated with empirical antibiotics, leading to antibiotic misuse and the development of antimicrobial resistance. The authors looked into 17 clinical and 42 immunological potential predictors for bacterial culture using a random forest or support vector machine (SVM) paired with recursive feature removal (RFE). The most effective clinical predictor to rule in and rule out UTI was urine cloudiness. Interestingly, adding the selected immunological biomarkers to the model with clinical features (including cloudiness or turbidity) did not improve the predictive properties. Dhanda et al. [14] described the NoMicro model, which does not take into account urine microscopy. Instead, the results of the urine dipstick test are used. Moreover, the authors generated NoMicro models based on several machine learning classificators, namely XGBoost, RF, and ANN, and compared their efficiency. The primary outcome was a pathogenic urine culture growing ≥ 100,000 colony-forming units. Predictor variables included age; gender; dipstick urinalysis nitrites, leukocytes, clarity, glucose, protein, and blood; dysuria; abdominal pain; and history of UTI. According to the results, the AUC of the NoMicro approach reached 0.85 in external validation and did not statistically differ from the version considering urine microscopy results. Arches et al. [15] described an application providing an analysis of the urine test strip using smartphones. According to the results, among the 65 participants, the confirmed UTI AI model achieved an overall accuracy rate of 96.03% and an overall reliability rate of ≥ 0.9, which is interpreted as excellent.

### Complicated UTI AI-Based Diagnosis

Research describing AI-based models for complicated UTI diagnosis is listed in Table 2. Møller et al. [16] aimed to develop two predictive models, using data from the index admission as well as historic data on a patient, to predict the development of UTI at the time of entry to the hospital and after 48 h of admission (HA-UTI). The ultimate goal was to assess the individual patient’s risk. The methodology included developing five machine learning models using features such as demographic information, laboratory results, past medical history, and clinical data. The unstructured features, such as the narrative text in electronic medical records, were preprocessed and converted to structured form by natural language processing. The area under the curve ranged from 0.82 to 0.84 for the entry model (*t* = 0 h) and 0.71 to 0.77 for the model predicting HA-UTI.

**Table 2:** Complicated UTI AI-based diagnosis

Author	Objective	Dataset (*n*)	AI model	Demographics	Anamnesis	Comorbidity	symptoms	Urine analysis	Imaging	Other	Performance	Validation
Møller et al. [16]	Prediction of UTI (model 1) and hospital-acquired UTI (model 2)	300,000	ANN	Age, gender	History of UTI	-	Fever, dysuria, frequency, urgency, suprapubic pain	-	-	-	AUC, 0.84	Internal
Taylor et al. [17]	Prediction of UTI in ED	80,387	XGBoost	Age, gender	History of UTI	-	Dysuria	Nitrites, WBC, RBC, bacteria, epithelial cells	-	-	AUC, 0.904 Sensitivity Specificity:	Internal
Mancini et al. [18]	Prediction of hospital-acquired multidrug-resistant UTI	1486	CatBoost	Age, gender	-	-	-	-	-	Ward, length of stay	AUC, 0.853 Sensitivity, 0.904	Internal
Cai et al. [19]	Prediction of clinical efficacy of antibiotics in women with recurrent UTIs	1043	ANN	-	Model 1: history of fluoroquinolones and cephalosporins failure, previously mentioned *E. coli* resistant to cotrimoxazole Model 2: previously mentioned *E. coli* resistant to cotrimoxazole and amoxicillin-clavulanic acid	-	-	-	-	-	For both: AUC, 0.87 Sensitivity, 87.8% Specificity, 97.3%	Internal
Chen et al. [20•]	Prediction of UTI after cystoscopy	1647	ANN	Age	History of UTI	-	-	-	Cystoscopy findings	-	Accuracy, 91% Sensitivity, 80% Specificity, 88%	Internal
Hong et al. [21]	Prediction of urosepsis among patients with upper urinary tract calculi	1716	ANN	Age, gender	-	Diabetes	Fever	WBC, nitrites, glucose	Ultrasound (hydronephrosis grade)	-	AUC, 0.95 Sensitivity, 80.4% Specificity, 98.2%	Internal

Taylor et al. [17] performed a single-center, multi-site, retrospective cohort analysis of adults who visited the emergency department based on urine culture results, clinical symptoms, and blood tests. Using both laboratory and clinical data, models for UTI prediction were created using six machine learning algorithms: RF, XGBoost, SVM, adaptive boosting, elastic net, and ANN. A full set of 211 variables and a reduced set of 10 variables (age, gender, history of UTI, dysuria, the presence of nitrites in urine, white blood cells (WBC), red blood cells (RBC), bacteria, and epithelial cells) were both used to develop the models. Comparisons between the UTI predictions and previously recorded UTI diagnoses were made. XGBoost, which has an area under the curve of 0.904, was found to be the best-performing method. It was also shown to have greater sensitivity when compared to the documentation of the UTI diagnosis. According to the results obtained, in practical application, approximately 1 in 4 patients will be re-classified from false positive to true negative, and 1 in 11 patients will be re-categorized from false negative to true positive on account of implementing the algorithm. Mancini et al. [18] created a machine learning model that can forecast a patient’s likelihood of developing a multidrug-resistant (MDR) UTI after being admitted to the hospital. The paper added a user-friendly cloud platform called DSaaS (Data Science as a Service), which is ideal for hospital organizations where healthcare operators might not have specialized programming language skills but need to analyze data, via machine learning techniques including CatBoost, SVM, and ANN. The paper employed DSaaS on a real antibiotic stewardship dataset. The development of an MDR UTI was predicted using data related to 1486 hospitalized patients, namely, sex, age, age class, ward, and time period. According to the results obtained, CatBoost exhibited the best predictive results, with the highest value in every metric used. Cai et al. [19] described two models based on ANN for predicting fluoroquinolone-based therapy failure (model 1) and fosfomycin-based therapy failure (model 2) among patients with recurrent UTI. Input data mostly consisted of previous urine culture profiles as well as types of antibiotic therapy failures. After the completion of the ANN learning and prediction processes, our neural network showed a sensitivity of 87.8% and a specificity of 97.3% in predicting the clinical efficacy of empirical therapy. Interestingly, the previous use of a specific class of antibiotic was not a risk factor for developing bacterial resistance to the same class (except for the fluoroquinolones), but instead, the most important risk factor for predicting resistance is the use of other classes of antibiotics.

Chen et al. [20•] compared models based on LR and ANN in defining UTI risk after cystoscopy to reduce antibiotic overuse. As input data, previous UTI history as well as cystoscopic findings such as benign prostatic hyperplasia (BPH), diverticulum, trabeculation, blood clot, cystocele, stone, and tumor was selected. The neural network model had a high accuracy of 85%, sensitivity of 80%, and specificity of 88%. Hong et al. [21] constructed a prediction model for urosepsis risk for patients with upper urinary tract calculi with the use of a machine learning ANN model. Several clinical and laboratory features, as well as a hydronephrosis degree based in the USA, were taken as predictive inputs. The area under the receiver operating curve in the validation set was 0.95. According to the results, the proposed model could provide risk assessments for urosepsis in patients with upper urinary tract calculi.

### UTI AI-Based Diagnosis in Susceptible Subgroups

Papers describing AI-based models for uncomplicated UTI diagnosis in susceptible subgroups are listed in Table 3. Pregnant women and children represent a separate subgroup of patients more susceptible to UTIs and requiring specific diagnostic flow and treatment. Pregnancy immunologic and urinary tract alterations predispose women to UTIs. Progesterone-induced smooth muscle relaxation and gravid uterine compression cause ureter and renal calyces dilatation. Also, vesicoureteral reflux may occur. These modifications exacerbate urinary tract infections [22]. In turn, UTIs are among the most prevalent bacterial pediatric infections. They are equally prevalent in males and girls during the first year of life but become more prevalent in girls following the first year [23]. This high susceptibility makes the development of decision support models based on AI even more relevant. Bertsimas et al. [24] developed a machine learning model to better stratify pediatric patients with vesicoureteral reflux complicated by UTI according to the effect of continuous antibiotic prophylaxis. The authors used the following data as input: vesicoureteral reflux grade, serum creatinine, race/gender, fever, dysuria, and weight, and achieved an AUC of 0.82. The described model allows better identification of patients for whom continuous antibiotic prophylaxis will be more effective, thereby providing a personalized approach, while minimizing use in those with the least need. A study by Burton et al. [25] aimed at introducing a way to increase the efficiency of urine culture results among pregnant women and children by reducing the number of query samples to be cultured and enabling diagnostic services to concentrate on those in which there are true microbial infections.

**Table 3:** UTI AI-based diagnosis in susceptible subgroups

Author	Objective	Dataset (*n*)	AI model	Demographics	Anamnesis	Comorbidity	Symptoms	Urine analysis	Imaging	Other	Performance	Validation
Bertsimas et al. [24]	Prediction of continuous antibiotic prophylaxis benefits among children with VUR and UTI	607	ANN	Race, gender, weight	-	-	Fever, dysuria	-	Ultrasound (vesicoureteral reflux grade)	Serum creatinine	AUC, 0.82	Internal
Burton et al. [25]	Prediction of urine culture in pregnant and children	212,554	XGBoost	Age, gender	History of urine culture results	-	-	WBC, bacteria	-	-	AUC, 91% Sensitivity, 95%	Internal
Zhu et al. [27]	Prediction of UTI among immobile stroke patients	7819	Ensemble learning model (ELM)	Age, gender	-	Pneumonia, mixed cerebrovascular disease	-	-	-	Length of stay, length of urethral catheterization, glucocorticoid use	AUC, 0.82 Sensitivity, 80.9%	External
Xu et al. [28]	Prediction of UTI among immobile stroke patients	3982	Siamese network	Age, gender	History of urethral invasive procedures	Classification of stroke, pneumonia, diabetes	Urine incontinence	-	-	Length of stay, duration of immobility, length of urethral catheterization, serum albumin	AUC: 0.83 Accuracy: 74.2% Sensitivity: 81% Specificity: 74%	Internal

This research discussed two methods of classification to test: one is a heuristic approach using a combination of features such as urine WBC and bacterial counts, and the second is testing typical machine learning models such as random forest, neural network, and XGBoost using independent features such as demographics, previous urine culture results, and clinical details as well. The most optimal solution found was three separate XGBoost algorithms trained separately for pregnant patients, children, and the rest of the categories. Combining the three models yielded a workload reduction of 41% and a sensitivity of 95% for each patient group. The work shows the possibility of using supervised machine learning models to improve service efficiency in situations where demand exceeds the number of resources available to public healthcare providers.

Immobile stroke patients also represent a highly susceptible patient subgroup. The prevalence of urinary tract infections is approximately 19%. In addition, the occurrence of an infection can exacerbate the physical harm caused by a stroke, forming a vicious circle with the stroke [26]. Zhu et al. [27] aimed to develop a prognostic model to define the risk of UTI among immobile stroke patients. Six machine learning models and an ensemble learning model were derived and evaluated. The latter achieved the best performance metrics both in internal and external validation sets, with an AUC of up to 0.82. Xu et al. [28] created an effective prediction model for identifying UTI risk in immobile stroke patients and compared its prediction performance to establish machine learning algorithms. They addressed this issue by developing a Siamese network that employed commonly used clinical criteria to identify patients at risk of UTIs. The model was developed and validated using a countrywide dataset of 3982 Chinese patients. A Siamese network is a deep neural network architecture with two or more identical subnetworks that are commonly employed in object detection. With an AUC of 0.83, the Siamese deep learning network did better than all the other machine learning–based models at predicting UTIs in stroke patients who were unable to move.

## Limitations and Future Directions

AI algorithms can identify unique correlations between symptoms, urinalysis results, and inflammatory processes in the urinary tract, as well as concise variable sets that are accurate in predicting urinary tract infections. Unquestionably, artificial intelligence is a highly precise and reliable instrument for predicting various events in healthcare [29]. In contrast to conventional statistics, artificial intelligence forecasts events by identifying distinct patterns. Sadly, along with new opportunities, associated difficulties with their application have emerged, necessitating a reduction in general optimism in order to comprehend the actual state of this technology, especially in the UTI field.

A sufficient amount of data is required for training neural networks to attain optimal performance metrics. In addition, limited dataset sizes may lead to estimation instability and overfitting [13]. According to our review, 10 of the 14 studies included more than 1000 cases, which, at first sight, may be an argument in favor of the utility of AI in the context of UTIs. In addition to quantity, however, the dataset must also be of sufficient quality. To be generalizable, the data should ideally be multicenter and prospectively collected, as well as span multiple geographic regions [14]. Furthermore, validation is an essential aspect of the reliability of the results. To obtain as objective and unbiased performance metrics as feasible, validation should be performed externally with samples that AI has never seen before [30]. Only two works provided external validation results in our review. On the other hand, the disparity in laboratory thresholds between medical centers and guidelines further complicates the collection of multicenter datasets and the routine application of the resulting AI-based models. For instance, there is currently no accepted level for a positive urine culture, with published values ranging from 10^2 to 10^5 cfu/mL. Conceivably different thresholds would result in different test performances [17].

The limitations outlined above represent only a small portion of the issues associated with the application of artificial intelligence and the interpretation of the results obtained. Despite this, the results of the studies included in this review demonstrate the potential utility of AI-based models for diagnosing UTIs. Clarifying the issues associated with the use of such technologies is an integral part of comprehending how the urological community should advance their sophistication. To facilitate the training of models, it is essential that as many medical centers around the world as possible converge on a common terminology for UTI, threshold values for various indicators, and research quality. To ensure generalizability, future studies should be prospective and multicenter to transition AI-based models from a stage of experimental development to a stage where they can be utilized in the clinical practice of urologists. Lastly, the advancement of AI in the field of UTIs is directly related to the general enhancement of diagnostic techniques. As new markers, new modalities, and improved interpretation become available, studies should be conducted to ascertain their utility in predicting UTIs using AI, thereby enhancing our knowledge of the future development of this technology.

There is great potential in using AI algorithms for the detection of urinary tract infections (UTIs), but there are also an array of challenges that need to be addressed. The investigations discussed demonstrate that machine and deep learning models have the potential to significantly improve UTI diagnosis, leading to faster, more precise diagnoses. The limitations and unknowns of their clinical influence, however, must be noticed. Although AI models have shown remarkable precision in specific settings, a wider variety of data is necessary to guarantee their consistency and generalizability. Larger datasets that comprise patients from many different backgrounds, ages, and locations fall under this category. To minimize errors and verify the efficacy of AI algorithms in actual clinical situations, prospective data collecting is essential. In addition, it is essential to acknowledge the value of external validation in making AI models robust and useful in various healthcare settings. Moving forward, healthcare facilities should work together to develop standardized diagnostic criteria and terminology for UTIs. This will help alleviate problems caused by disparities in laboratory methods and terminology. This will allow AI algorithms to be more seamlessly integrated into the diagnostic workflow, minimizing the need for intrusive and expensive laboratory testing and imaging.

## Conclusion

AI-driven UTI detection is a promising new area of healthcare research; however, it is still in the exploratory rather than implementation phase. To fully realize AI’s potential for enhancing UTI diagnosis, more study is needed, ideally guided by larger, more diversified datasets and rigorous validation techniques. By resolving these issues, we can bring AI to bear on this important aspect of healthcare, which will improve patient care, cut costs, and slow the spread of antibiotic resistance. Further studies utilizing large, heterogeneous, prospectively collected datasets, as well as external validations, are required to define the actual clinical workflow value of artificial intelligence.

## Data Availability

Data is available on request.
